# Quercetin Dietary Supplementation Advances Growth Performance, Gut Microbiota, and Intestinal mRNA Expression Genes in Broiler Chickens

**DOI:** 10.3390/ani11082302

**Published:** 2021-08-04

**Authors:** Mervat A. Abdel-Latif, Ahmed R. Elbestawy, Ali H. El-Far, Ahmed E. Noreldin, Mohamed Emam, Roua S. Baty, Ghadeer M. Albadrani, Mohamed M. Abdel-Daim, Hatem S. Abd El-Hamid

**Affiliations:** 1 Department of Nutrition and Veterinary Clinical Nutrition, Faculty of Veterinary Medicine, Damanhour University, Damanhour 22511, Egypt; vetdr_2006@yahoo.com; 2 Department of Poultry and Fish Diseases, Faculty of Veterinary Medicine, Damanhour University, Damanhour 22511, Egypt; ahmed.elbestawy@vetmed.dmu.edu.eg (A.R.E.); drhatem_deltavet@yahoo.co.uk (H.S.A.E.-H.); 3 Department of Biochemistry, Faculty of Veterinary Medicine, Damanhour University, Damanhour 22511, Egypt; ali.elfar@damanhour.edu.eg; 4 Histology and Cytology Department, Faculty of Veterinary Medicine, Damanhour University, Damanhour 22511, Egypt; ahmed.elsayed@damanhour.edu.eg; 5 Department of Biotechnology, College of Science, Taif University, P.O. Box 11099, Taif 21944, Saudi Arabia; rsbaty@tu.edu.sa; 6 Department of Biology, College of Science, Princess Nourah bint Abdulrahman University, Riyadh 11474, Saudi Arabia; gmalbadrani@pnu.edu.sa; 7 Department of Pharmaceutical Sciences, Pharmacy Program, Batterjee Medical College, P.O. Box 6231, Jeddah 21442, Saudi Arabia; 8 Pharmacology Department, Faculty of Veterinary Medicine, Suez Canal University, Ismailia 41522, Egypt

**Keywords:** antioxidant, broilers, growth performance, gut microbiota, quercetin

## Abstract

**Simple Summary:**

The biological activity of quercetin is diverse, particularly antioxidant, antimicrobial, and antibacterial. The impacts of quercetin nutritional supplementations on growth performance, humoral immunity, gut microbiota and mRNA in broiler chickens were recorded.

**Abstract:**

Quercetin was fed to groups of broiler chickens at concentrations of 200, 400, and 800 ppm, and a control group was supplemented with a basal diet. Results revealed that quercetin dietary supplementation numerically improved the growth performance traits and significantly increased (*p* < 0.05) the European production efficiency factor (EPEF) in the 200 ppm group. The total coliforms and *Clostridium perfringens* were decreased (*p* < 0.05) in quercetin-supplemented groups. Conversely, *Lactobacillus* counts were increased (*p* < 0.05), due to improvement of the gut microbiota environment in quercetin-supplemented groups. Moreover, the mRNA expression of intestinal Cu/Zn-superoxide dismutase (*SOD1*), glutathione peroxidase (*GSH-Px*) and nutritional transporters, including glucose transporter 2 (*GLUT2*), peptide transporter 1 (*PEPT1*), and fatty acid synthase (*FAS*) genes, were significantly upregulated in quercetin-supplemented groups. Quercetin enhanced intestinal morphometry. We can suggest quercetin supplementation in broiler chickens by levels between 200 and 400 ppm to enhance their development and gut environment.

## 1. Introduction

The microbiome plays a major role in the gastrointestinal tract health, immune system, and productivity of broiler chickens [1]. The link between intestinal health and overall health is raised by the quality of feed consumed by broiler chickens [2]. Many studies have been conducted to enhance the intestinal environment by adding herbs, probiotics, and exogenous enzymes to the diet of broiler chickens to increase their efficiency and productivity [3,4,5,6,7,8].

The global banning of antibiotic growth promotors has encouraged researchers to find alternative solutions. Flavonols can regulate feed intake, contribute to eubiosis, and exhibit antimicrobial, immunomodulatory, anti-inflammatory, and antioxidant properties in monogastric animals [9]. Quercetin (3,3′,4′,5,7-pentahydroxyflavone) is a constituent of flavonols, a sub-group of flavonoids that are present in some fruit (apples, berries, and grapes), herbs, and some vegetables (onions and broccoli) [10]. Quercetin is a powerful, anti-inflammatory, antimicrobial, anti-obesity, anti-hypercholesterolemic, antioxidant, anti-aging, and anticancer agent [11]. Having antioxidative action is the primary core of quercetin’s biological activities. Quercetin is generally one of the most commonly used bioflavonoids for metabolic and inflammatory diseases [12]. 

Regarding the role of quercetin in broiler diets, the study of Goliomytis et al. [13] stated that quercetin may extend the shelf-life of meat by decreasing the lipid oxidation rate and may lead to enhanced animal health. Quercetin also increases the immune responses in broiler chickens [14]. The current research examines the impact of various quercetin supplementation concentrations on the performance, gut microbiota, humoral immunity, and some mRNAs of broiler chickens.

## 2. Materials and Methods

### 2.1. Ethics Statement 

The research was endorsed by the Local Experimental Animal Care Committee of the University of Damanhour, Egypt, Faculty of Veterinary Medicine (VMD: 15/2018).

### 2.2. Animals, Management and the Experimental Design

One hundred and twenty-eight one-day-old Ross 308 chicks were obtained from a commercial hatchery and randomly allocated into four equal groups of mixed-sex chicks (32 birds of equal body weights per group (41 gm/chick)). Each group was subdivided into four replicates (8 birds per replicate) with an equal number of males in each replicate (2 males and 6 females) and raised on wire-floored cages of the same dimensions, and numbers of nipple drinkers and feed hoppers. They received an experimental diet for five consecutive weeks. The birds were allowed to consume feed and water ad libitum and were kept under daily observation. The environmental temperature of the 1st week was 32 °C and progressively reduced to 26 °C by the 3rd week of age, and the chicks were exposed to 23 h light. Chicks were allocated into control group 1 (control, fed on a commercial basal diet); group 2 (Q200), group 3 (Q400), and group 4 (Q800) were fed a commercial basal diet containing 200, 400, and 800 ppm quercetin (Sigma-Aldrich Chemical Co., St. Louis, MO, USA), respectively. The basal diets (corn–soybean based diet) were formulated according to the nutrient requirements for broiler chickens (Ross 308) [15]. The nutrient content of the ingredients was evaluated following the instructions of AOAC [16]. The ingredients’ percentage and calculated composition analysis of the basal diet are shown in Table 1. 

Broiler chickens were vaccinated under the following program in all cages: inactivated avian influenza subtype H5N1 vaccine (MeFluvac^®^, MEVAC, Cairo, Egypt) by subcutaneous injection, and bivalent live Newcastle disease and infectious bronchitis vaccine (Nobilis^®^ Clone 30 + Ma5, MSD, Boxmeer, The Netherlands) at 7 days of age; live Gumboro intermediate plus (Bursine Plus^®^ vaccine, Zoetis Inc., Florham Park, NJ, USA) at 14 days of age, and finally vaccinated with live Newcastle disease (Nobilis^®^ ND LaSota, MSD, Boxmeer, The Netherlands) vaccine at 18 days of age. Eye drops were used to administer all live vaccines.

### 2.3. Growth Performance 

The performance parameters include the body weight (BW), feed intake (FI), body weight gain (BWG), feed conversion ratio (FCR), and protein efficiency ratio (PER). The viability percentage was assessed weekly over the whole experimental period. In addition, the European production efficiency factor (EPEF) was evaluated at days 21 and 35 of the study [17] and calculated according to the following equation:

EPEF = [(viability% × body weight per kg) ÷ (age per day× FCR)] × 100(1)

### 2.4. Sample Collection 

At days 21 and 35, fecal samples were collected and tested for total coliform, *C. perfringens* and *Lactobacillus* counts. At the end of the experiment, day 35, blood samples (n = 5) were collected from the wing vein without anticoagulant for serum separation. The collected samples were centrifuged at 1435× g for 15 min at 4 °C to obtain clear sera for HI test against avian influenza subtype H5N1. Five birds from each group were euthanized through anesthesia with intravenous injection of sodium pentobarbital (50 mg/kg) and immediately necropsied. Five intestinal (ileum) samples of 2 cm in length (5 cm proximal to the ileo—cecal junction) for mRNA gene expressions and histological analysis were taken from each group, processed, and analyzed as previously described [5].

### 2.5. Total Fecal Bacterial Count 

The total coliform, *C. perfringens*, and *Lactobacillus*, counts were evaluated as previously described [5].

Briefly, ten-fold dilutions (10 ^−1^ to 10 ^−7^) of each sample were performed with BPW and directly inoculated on MacConkey’s agar for total coliform counting and incubated aerobically at 37 °C for 24 h. All red colonies within the range of 15–150 μm were selected for counting.

*C. perfringens* were subcultured on Perfringens agar base (Oxoid; Table 2) mixed with 400 mg of D-cycloserine per liter by the dilutions from 10 ^−1^ to 10 ^−7^ and incubated anaerobically at 37 °C, using gas generating kits (Oxoid) for 48 h. Plates with black colonies within the range of 25–250 μm were counted.

The *Lactobacillus* count was conducted using Rogosa agar (Table 3) plates and cultured by dilutions from 10 ^−1^ to 10 ^−7^, then incubated at 37 °C in 5% CO_2_. All whitish colonies that appeared after 48 h of incubation were counted.

### 2.6. Hemagglutination Inhibition (HI) Assay

Antibody titers for avian influenza subtype H5N1 were determined using a standard H5N1 antigen; the positive titers were the highest dilutions of serum causing complete inhibition of 4 hemagglutination units (4 HAU) of antigen [18,19].

### 2.7. RNA Extraction and RT-PCR

The total RNA was extracted and purified from intestinal samples (n = 5) of all groups using QIAamp RNeasy Mini kit (Qiagen, GmbH, Hilden, Germany); then, RT-PCR was done with QuantiTect SYBR Green PCR Master Mix (Qiagen, GmbH, Dusseldorf, Germany). Primers are listed in Table 4 as previously practiced [5].

### 2.8. Histology

The fixed samples were processed with the conventional paraffin embedding technique and stained with hematoxylin and eosin (H&E) as described by Bancroft and Layton [20]. Sectioning and slide preparation were done according to Saeed et al. [21]. Three sections were utilized from each intestinal segment (one section from serial ten sections). From every section, five well oriented complete villi were selected for the investigation. So, fifteen values were estimated for each intestinal sample. Slides were examined under a light microscope (Leica DM500) at 4× magnification, using a digital camera (Leica EC3, Leica, Germany). Measurements of the villi height (VH), villi width in the middle of the individual villus (VW), crypt depth (CD), and VH:CD ratio for each villus in the control and supplemented groups were made by using ImageJ software (NIH, Bethesda, MD, USA) [22].

### 2.9. Statistical Analysis

Statistical calculations were made with the SPSS programming tool (IBM SPSS. 20^®^) (SPSS Inc., Chicago, IL, USA) using one-way ANOVA followed by Duncan’s multiple range tests. Data of the HI assay, RT-PCR, and total fecal bacterial counts were analyzed with one-way ANOVA and Tukey’s multiple range tests with Graphpad prism 5. All significant deviations were based on *p* < 0.05.

## 3. Results

### 3.1. Growth Performance and Survival Percentages

The initial live body weight between the distinct experimental groups non-significantly varied (Table 5). In Q200, Q400, and Q800, growth was enhanced during the experimental period by quercetin supplementation, compared with the control, by 3.27, 3.18, and 2.32%, respectively, and feed intake values were similar to the control group. In comparison with the control groups, the body weight gain, feed conversion ratios (FCR), and protein efficiency ratio (PER) values were not substantially improved; however, the European production efficiency factor (EPEF) was increased (*p* < 0.05) in Q200 as compared with the control group. In addition, the quercetin-supplemented groups expressed no mortality, compared to 3.13% of the control.

### 3.2. Total Fecal Bacterial Count 

**Figure 1:**
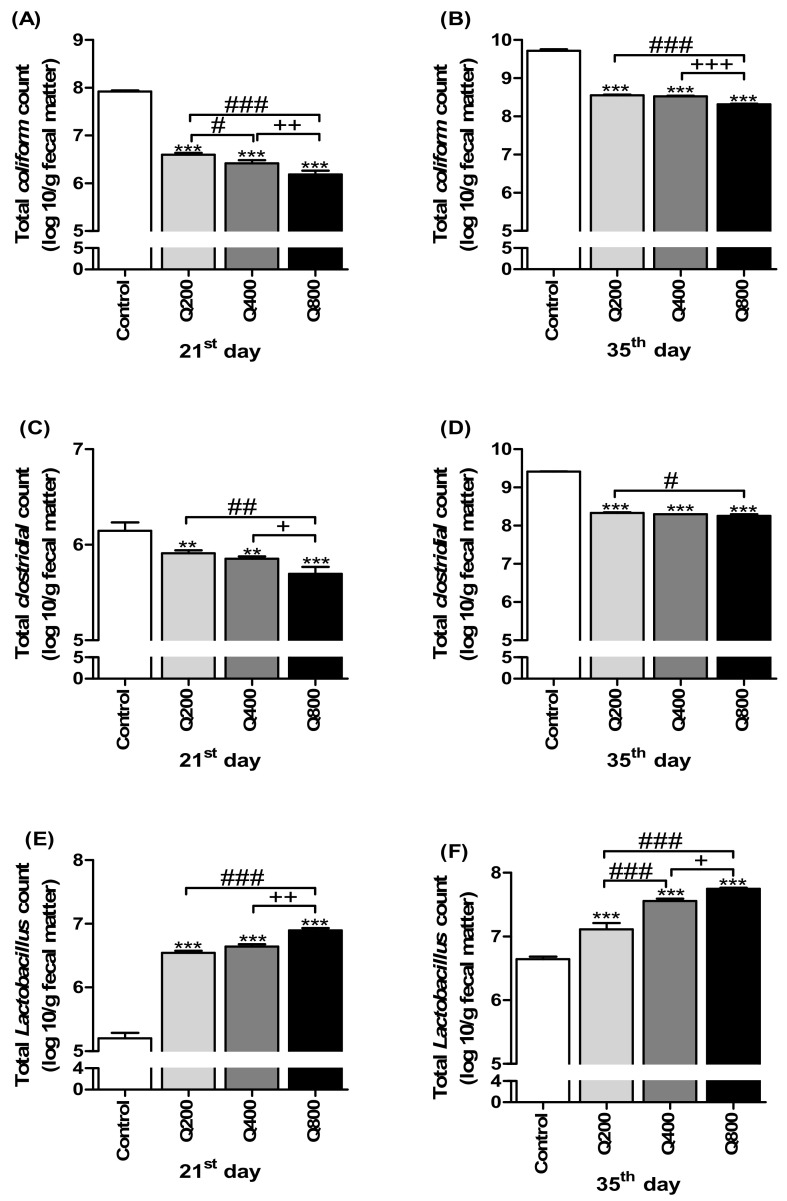
Total coliform (**A,B**), *C. perfringens* (**C,D**), and *Lactobacillus* (**E,F**) counts in fecal samples. ** *p* < 0.01 and *** *p* < 0.001 vs. control. ^#^ *p* < 0.05, ^##^ *p* < 0.01, and ^###^ *p* < 0.001 vs. Q200. ^+^ *p* < 0.05, ^++^ *p* < 0.01, and ^+++^ *p* < 0.001 vs. Q400. Statistical analysis was performed using one-way ANOVA and Tukey’s post hoc test for multiple comparisons. Q200, birds fed 200 ppm. Q400, birds fed 400 ppm. Q800, birds fed 800 ppm.

Total coliform counts decreased significantly (*p* < 0.001) by 21 and 35 days in all quercetin-supplemented groups (Q200, Q400, and Q800) (Figure 1A,B), compared with the control group. Total *Clostridium perfringens* counts were decreased in all quercetin supplements (*p* < 0.001) at 35 days of age (9.41 log10 CFU/g), compared with the control (Figure 1C,D). Conversely, the total number of *Lactobacillus* in groups supplemented with quercetin was enhanced (*p* < 0.001), compared with the control at both ages (21 and 35 days) (Figure 1E,F).

### 3.3. Hemagglutination Inhibition Test

**Figure 2:**
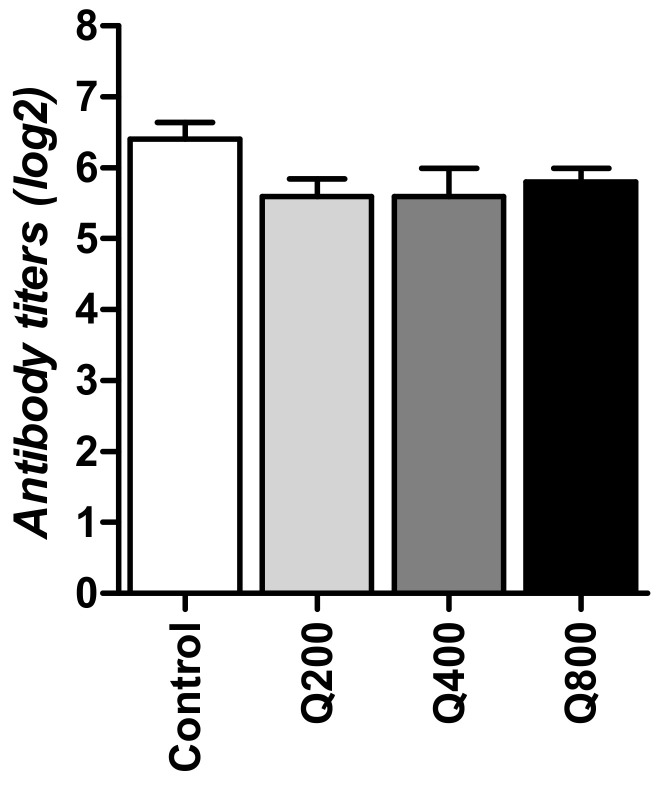
Antibody titer of H5N1 (log2). Statistical analysis was performed using one-way ANOVA and Tukey’s post hoc test for multiple comparisons. Q200, birds fed 200 ppm. Q400, birds fed 400 ppm. Q800, birds fed 800 ppm.

In the quercetin-supplemented groups and controls, no significant differences were found among HI Titer values of the H5N1 avian influenza subtype; however, the titers of Q200, Q400, and Q800 were below the control level at 5.4, 5.4, 5.8 vs. 6.4 log2, respectively (Figure 2).

### 3.4. Antioxidant Enzymes’ Gene Expressions

**Figure 3:**
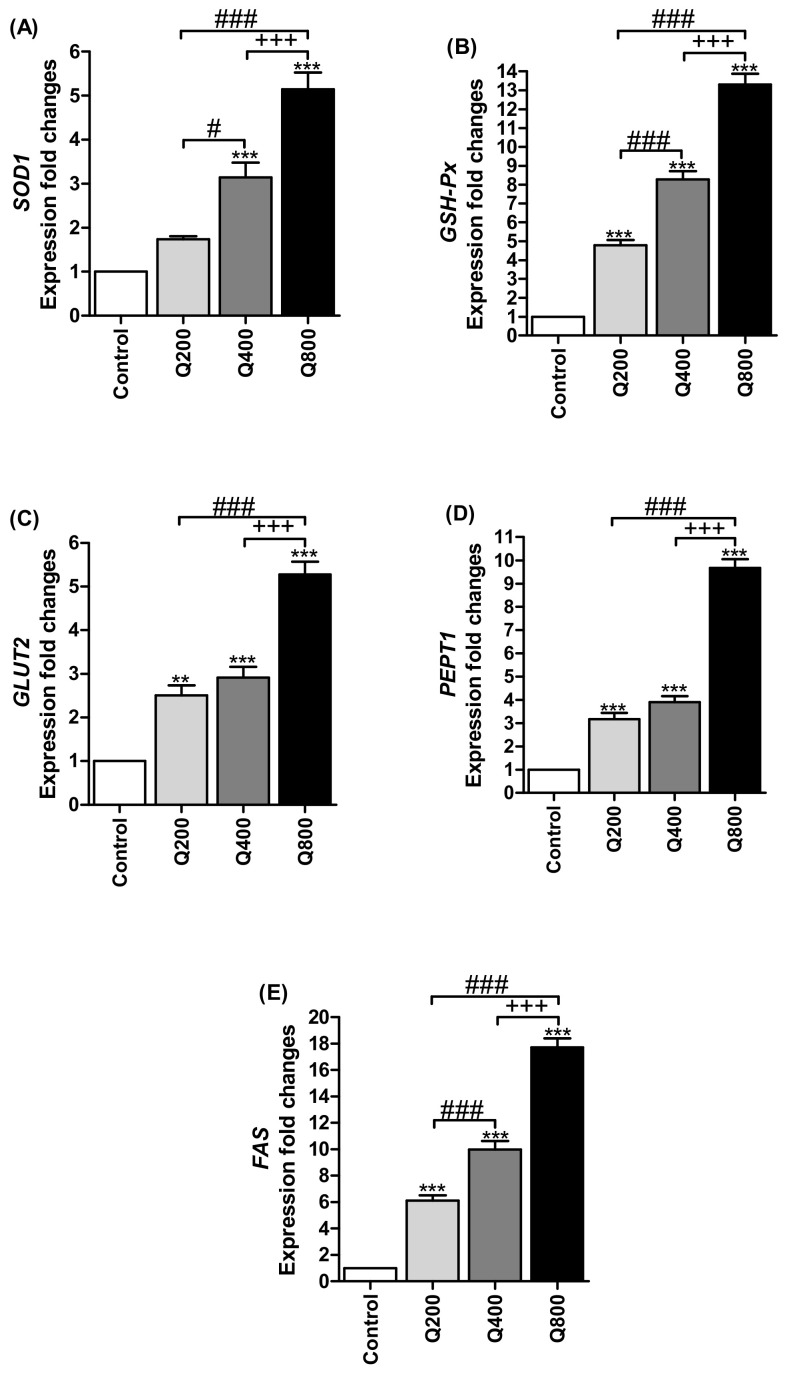
RT-PCR validation of the *SOD1* (**A**)*, GSH-Px* (**B**), *GLUT2* (**C**)*, PEPT1* (**D**), and *FAS* (**E**) genes. ** *p* < 0.01 and *** *p* < 0.001 vs. control. ^#^ *p* < 0.05 and ^###^ *p* < 0.001 vs. Q200. ^+++^ *p* < 0.001 vs. Q400. Statistical analysis was performed using one-way ANOVA and Tukey’s post hoc test for multiple comparisons. Q200, birds fed 200 ppm. Q400, birds fed 400 ppm. Q800, birds fed 800 ppm.

As shown in Figure 3A, the expressions of the intestinal Cu/Zn-superoxide dismutase (SOD1) in the Q400 and Q800 groups were significantly increased (*p* < 0.001), compared with the control group, and were higher (*p* < 0.05 and *p* < 0.001, respectively) than Q200. Moreover, the SOD1 gene expression was increased (*p* < 0.001) in Q800, compared with Q400. Data in Figure 3B show the mRNA expression of glutathione peroxidase (GSH-Px); the quercetin-supplemented groups had higher (*p* < 0.001) levels than the control. The fold change of GSH-Px expression in Q800 and Q400 was increased (*p* < 0.001), compared with Q200, while Q800 was also increased (*p* < 0.001) in comparison with Q400.

### 3.5. Nutrients Transporter Gene Expressions

Figure 3C,D shows the expression of intestinal glucose transporter 2 (GLUT2) and peptide transporter 1 (PEPT1), respectively. GLUT2 and PEPT1 expressions were increased (*p* < 0.001) in quercetin-supplemented groups, compared with the control group. Additionally, their expressions increased (*p* < 0.001) in Q800, compared with Q400 and Q200. The same results were found for the intestinal fatty acid synthase (FAS) gene (Figure 3E) with a significant increase (*p* < 0.001) in Q400, compared with the Q200 group.

### 3.6. Histology 

**Figure 4:**
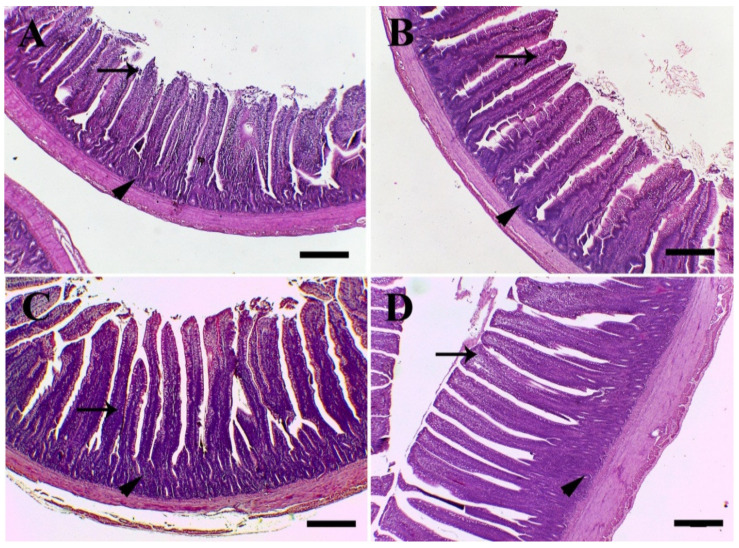

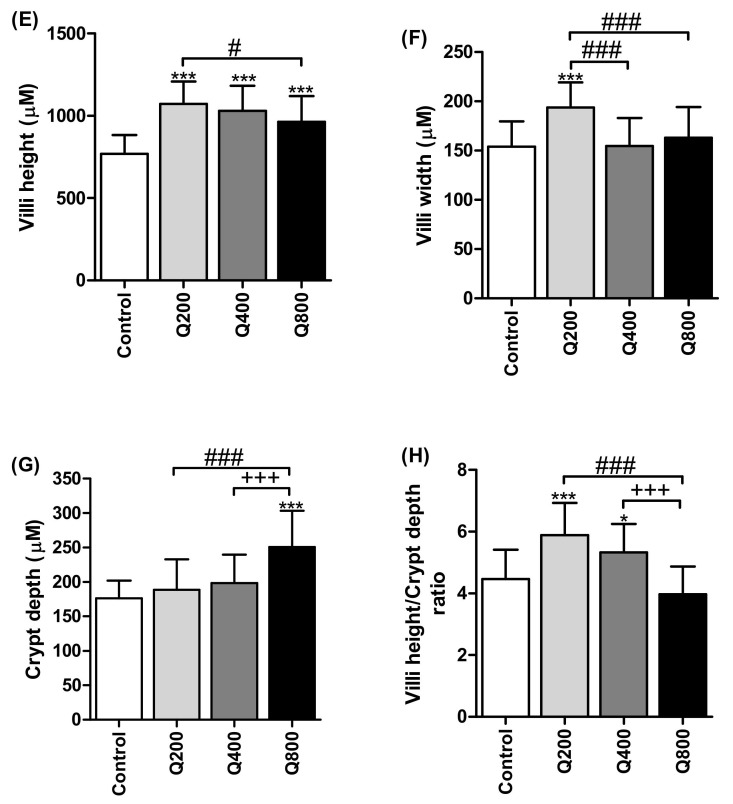
Light microscopic images of small intestine. (**A**) Control. (**B**) Q200 shows the highest villi height and width and the lowest crypt depth. (**C**) Q400 shows the moderate villi height, width and crypt depth. (**D**) Q800 shows the lowest villi height and width and the greatest crypt depth. (**E**) Villi height (µm). (**F**) Villi width (µm). (**G**) Crypt depth (µm). (**H**) Villi height/crypt depth. * *p* < 0.05 and *** *p* < 0.001 vs. control. ^#^ *p* < 0.05 and ^###^ *p* < 0.001 vs. Q200. ^+++^ *p* < 0.001 vs. Q400. Statistical analysis was performed using one-way ANOVA and Tukey’s post hoc test for multiple comparisons. Q200, birds fed 200 ppm. Q400, birds fed 400 ppm. Q800, birds fed 800 ppm. The black arrows refer to villi. The black arrows’ heads refer to crypts. Scale bar ₌ 400 µm.

The height and width of the villi in the quercetin-supplemented groups compared with the control group significantly increased. The best supplementation for the villi area was Q200. On the other hand, Q800 had the best effect on the crypt depth. Villi height/crypt depth had the highest value in the Q200 group, due to the highest effect of this supplementation on the villi (Figure 4).

## 4. Discussion

Quercetin is a bioavailable glycone in mammals. Quercetin glycosides can be hydrolyzed by lactase phlorizin hydrolase in the lumen or, once entered into the enterocyte through the sodium dependent glucose transporter (SGLT1), quercetin–glucoside is hydrolyzed by β-glucosidase [27,28,29]. In addition, in chickens, Rupasinghe et al. [30] detected seven quercetin metabolites in the excreta of quercetin-supplemented birds, indicating that quercetin was absorbed and excreted. Alternatively, the presence of metabolites in broiler chicken excreta could be due to the action of intestinal microbiota, rather than de novo metabolism. Other studies confirmed the absorption of quercetin in pigs [31] and horses [32].

Functional feeds are used to improve poultry efficiency by regulating pathogens and improving beneficial bacteria in the intestines [33]. This results in improved weight gain, FCR, and uniformity [34]. Here, quercetin improved performance parameters, such as body weight (BW), body weight gain (BWG), FCR, PER, and EPEF, while voluntary feed intake (VFI) in the quercetin-supplemented groups did not differ significantly from the control group. In the Q200 group, EPEF increased noticeably, compared with the control group. In the same context, Liu et al. [35] supplemented hens with varying quercetin levels and found an enhanced laying rate and FCR when hens were fed 0.2 and 0.4 g quercetin per kg of body weight. 

The improvement of performance parameters in our research was attributed to the metabolic prebiotic effect of quercetin [35] with its ability to modulate gut microbiota through increasing the number of beneficial bacteria as Lactobacilli and decreasing the number of *C. perfringens* and total coliform counts (selective action) that beneficially affect the broiler chickens’ health and performance. Quercetin induces their antibacterial activity by acting as DNA gyrase on various cell targets [36], bacterial membrane and motility [37], type II fatty acid biosynthesis (FAS II) pathway [38], and D-alanine:d-alanine ligase (Ddl) enzyme inhibitor, acting as a bacteriostatic, preventing harmful bacterial growth [39]. Unfortunately, the fecal materials were collected without caecum voiding, so there was no differentiation between fecal and cecal droppings [40]; however, this is a very critical limitation point and it will be considered in future work.

Villus height and crypt depth from the jejunum of the quercetin fed birds increased, compared with the control. The height of villi shows the gut’s absorbing ability [41]. The supplemented treatment deviated significantly from the control in the villus height and villus/crypt ratio in the jejunum at Q200, although the villus/crypt ratio of the birds fed Q800 tended to be lower than other treatments, compared with the control. These results indicate that feeding quercetin to broilers may boost intestinal morphology, which shows elevated absorption and enhanced intestinal health. Similarly, in rats, the administration of methotrexate-treated quercetin resulted in a higher villus height in the jejunum and ileum crypts [42]. Moreover, quercetin dietary supplementation upregulated the expression of nutrient transporter genes (GLUT2, PEPT1, and FAS), which play a vital role in the nutrient’s metabolism. Intestinal GLUT2 is primarily a protein sensor for glucose and glucose homeostasis [43]. In the absorption of small peptides, the intestinal PEPT1 plays a key role [44], while the intestinal FAS retains the palmitoylation of the mucin 2, intestinal mucus barrier, which prevents the bowel pathogen [45]. In addition, the status of the bowel antioxidant is a key protection indicator for broiler chickens. In the present study, SOD1 and GSH-Px mRNA expressions in quercetin-supplemented groups were significantly increased dose-dependently, compared with the control. SOD enzymes catalyze the transformation of superoxide anion into less dangerous free radical hydrogen peroxide (H_2_O_2_) [46], and GSH-Px attacked the generated H_2_O_2_ [47]. These antioxidant effects of quercetin provided the birds with a strong defense with a healthy intestinal environment [5]. Iskender et al. [48] reported significant increases in antioxidant enzymes, including the activities of GSH-Px, SOD1, and glutathione levels in erythrocytic lysates of laying hens fed a diet containing 0.5 g/kg quercetin.

Immune response to vaccination is a key method to show the well-functioning of the immune system of broiler chickens [49]. Here, we investigated the impact of quercetin supplementation on the healthy intestinal immunity and, accordingly, on the overall humoral immune response, e.g., serological immune response to inactivated avian influenza vaccine (H5N1) at the end of the experiment; the quercetin-supplemented groups did not differ significantly in relation to controls. However, intestinal injury models are badly needed in the future to explore the close relationship between the gastrointestinal tract (GIT) microflora and development and/or maintenance of a functional intestinal immune system during quercetin supplementation.

## 5. Conclusions 

Quercetin dietary supplementation in broiler chickens of 200 and 400 ppm, but not 800 ppm, enhanced their growth, intestinal and gene expression in levels of antioxidant enzymes and nutrient transportation. Quercetin is, therefore, regarded as a promising natural feed additive to broiler chickens at levels below 800 ppm.

## Figures and Tables

**Table 1:** Ingredients’ percentage and calculated composition analysis of the experimental starter and grower diets (%, as-fed basis).

Ingredients %	Starter (0–10 d)	Grower (11–21 d)	Finisher (22–35 d)
Yellow corn	54.78	58.88	63.90
Soybean meal (44%)	33.5	29.4	24
Corn gluten (60%)	5	5	5
Corn oil	2	2.65	3.15
Dicalcium phosphate	1.73	1.6	1.5
Lime stone	1.35	1	1
Salt	0.4	0.4	0.4
DL-methionine *	0.15	0.12	0.1
HCl-lysine **	0.35	0.3	0.3
Vitamins and minerals premix ***	0.3	0.3	0.3
Antimycotoxin	0.2	0.2	0.2
Sodium bicarbonate	0.1	0.1	0.1
Choline chloride	0.05	0.05	0.05
Calculated composition			
ME, Kcal/Kg diet	3005	3100	3195
CP%	23	21.5	19.5
Ca%	1	0.87	0.82
Avail. P%	0.47	0.44	0.41
Methionine%	0.56	0.51	0.47
Lysine%	1.44	1.29	1.14
Meth. + Cyst.%	0.93	0.86	0.78
Na%	0.20	0.20	0.20

SBM = soybean meal, ME = metabolizable energy, CP = crude protein, Av. (P) = available phosphorous. * DL—methionine 99% feed grade China. ** L—lysine 99% feed grade. *** Vitamin and mineral premix (Hero mix) produced by Hero pharm and composed (per 3 kg) of vitamin A 12,000,000 IU, vitamin D3 2,500,000 IU, vitamin E 10,000 mg, vitamin K3 2000 mg, vitamin B1 1000 mg, vitamin B2 5000 mg, vitamin B6 1500 mg, vitamin B12 10 mg, niacin 30,000 mg, biotin 50 mg, folic acid 1000 mg, pantothenic acid 10,000 mg, manganese 60,000 mg, zinc 50,000 mg, iron 30,000 mg, copper 4000 mg, iodine 300 mg, selenium 100 mg, and cobalt 100 mg.

**Table 2:** Composition of Perfringens agar.

Ingredients	Amount (g)
Tryptose	15.0
Soya peptone	5.0
Yeast extract	5.0
Sodium metabisulphite	1.0
Ferric ammonium citrate	1.0
Agar	19.0
Distilled water added to make 1 liter	

**Table 3:** Composition of Rogosa agar.

Ingredients	Amount (g)
Tryptone	10.0
Yeast extract	5.0
Glucose	20.0
Sodium acetate, anhydrous	17.0
Ammonium citrate	2.0
Potassium dihydrogen phosphate	6.0
Magnesium sulfate	0.575
Manganese sulfate	0.120
Ferrous sulfate	0.034
Bacteriological Agar	20
Tween 80	1
Distilled water added to make 1 liter	

**Table 4:** Primer sequences, target genes, amplicon sizes and cycling conditions for SYBR green RT-PCR.

Target Gene	Primers Sequences	Reverse Transcription	Primary Denaturation	Amplification (40 Cycles)			Dissociation Curve (1 Cycle)			Reference
				Secondary Denaturation	Annealing (Optics on)	Extension	Secondary Denaturation	Annealing	Final Denaturation	
*β. actin*	F: ATTGTCCACCGCAA ATGCTTC	50 °C 30 min	94 °C 5 min	94 °C 15 s	60 °C 30 s	72 °C 30 s	94 °C 1 min	60 °C 1 min	94 °C 1 min	[23]
	R: AAATAAAGCCATGCCAATCTCGTC									
*SOD1*	F: AGGGGGTCATCCACTTCC				60 °C 30 s			60 °C 1 min		[24]
	R: CCCATTTGTGTTGTCTCCAA									
*GSH-PX*	F: TTGTAAACATCAGGGGCAAA									
	R: ATGGGCCAAGATCTTTCTGTAA									
*GLUT2*	F: CACACTATGGGCGCATGCT				60 °C 30 s			60 °C 1 min		[25]
	R: ATTGTCCCTGGAGGTGTTGGTG									
*PEPT1*	F: CCCCTGAGGAGGATCACTGTT									
	R: CAAAAGAGCAGCAGCAACGA									
*FAS*	F: CTATCGACACAGCCTGCTCCT				62 °C 30 s			62 °C 1 min		[26]
	R: CAGAATGTTGACCCCTCCTACC									

**Table 5:** Effect of dietary quercetin supplementation on growth performance and mortality rate of broilers.

	Control	Quercetin Supplementation			
		Q200	Q400	Q800	*p-Value*
Initial weight, g	41.46 ± 0.47	41.67 ± 0.49	41.67 ± 0.49	41.67 ± 0.49	0.99
^1^ fBwt, g	1768.10 ± 31.56	1826.59 ± 35.88	1824.29± 31.97	1809.13 ± 25.77	0.54
^2^ BWG, g	1727.14 ± 31.36	1785 ± 35.58	1782.86 ± 31.60	1767.61 ± 25.45	0.54
BWG/day, g	49.35 ± 0.90	51 ± 1.02	50.94 ± 0.90	50.50 ± 0.73	0.54
^3^ FI, g	2887.24± 25.32 ^ab^	2848.31 ± 28.45 ^b^	2894.72 ± 6.11 ^ab^	2926.58 ± 11.29 ^a^	0.05
^4^ FCR	1.67 ± 0.03	1.60 ± 0.033	1.63 ± 0.03	1.66 ± 0.02	0.32
^5^ PER	2.80 ± 0.05	2.93 ± 0.06	2.87 ± 0.05	2.81 ± 0.04	0.27
^6^ EPEF	296.54 ± 10.12 ^b^	331.06 ± 13.03 ^a^	323.29 ± 11.54 ^ab^	313 ± 8.32 ^ab^	0.01
Mortality%	3.13	0	0	0	

Note: Means within each column for each division with no common superscript letters are significantly different (*p* ≤ 0.05). Abbreviations. ^1^ Final body weight. ^2^ Body weight gain. ^3^ Voluntary feed intake. ^4^ Feed conversion ratio. ^5^ Protein efficiency ratio. ^6^$\;\mathrm{EPEF}=\left[\left(\mathrm{viability}\;\%\;\times \;\mathrm{body}\;\mathrm{weight}\;\mathrm{per}\;\mathrm{kg}\right)\;÷\left(\mathrm{age}\;\mathrm{per}\;\mathrm{day}\times \;\mathrm{FCR}\right)\right]\times 100$.

## Data Availability

The data presented in this study are available on request from the corresponding author.
